# Chemical Characterization and Determination of the Antioxidant Properties of Phenolic Compounds in Three *Scutellaria* sp. Plants Grown in Colombia

**DOI:** 10.3390/molecules28083474

**Published:** 2023-04-14

**Authors:** Silvia M. Porras, Rogerio A. Saavedra, Lady J. Sierra, Robert T. González, Jairo R. Martínez, Elena E. Stashenko

**Affiliations:** 1 Research Center for Chromatography and Mass Spectrometry (CROM-MASS), Center for Biomolecules (CIBIMOL), Universidad Industrial de Santander, Carrera 27, Calle 9, Bucaramanga 680002, Colombia; silvia2208463@correo.uis.edu.co (S.M.P.); cenivam4@tucan.uis.edu.co (R.A.S.); lady.sierra2@correo.uis.edu.co (L.J.S.); rene@tucan.uis.edu.co (J.R.M.); 2 Research Group on Orchids and Ecology, Universidad Nacional de Colombia, Carrera 32, Palmira 763533, Colombia; rtgonzalezm@unal.edu.co

**Keywords:** *Scutellaria*, incarnata, coccinea, UHPLC/HRMS, antioxidant activity

## Abstract

Plants of the genus *Scutellaria* (Lamiaceae) have a wide variety of bioactive secondary metabolites with diverse biological properties, e.g., anti-inflammatory, antiallergenic, antioxidant, antiviral, and antitumor activities. The chemical composition of the hydroethanolic extracts, obtained from dried plants of *S*. *incarnata*, *S*. *coccinea*, and *S*. *ventenatii* × *S*. *incarnata*, was determined by UHPLC/ESI-Q-Orbitrap-MS. The flavones were found in a higher proportion. Baicalin and dihydrobaicalein-glucuronide were the major extract components in *S*. *incarnata* (287.127 ± 0.005 mg/g and 140.18 ± 0.07 mg/g), in *S*. *coccinea* (158.3 ± 0.34 mg/g and 51.20 ± 0.02 mg/g), and in *S*. *ventenatii* × *S*. *incarnata* (186.87 ± 0.01 mg/g and 44.89 ± 0.06 mg/g). The *S*. *coccinea* extract showed the highest antioxidant activity in the four complementary techniques employed to evaluate all extracts: ORAC (3828 ± 3.0 µmol Trolox^®^/g extract), ABTS^+•^ (747 ± 1.8 µmol Trolox^®^/g extract), online HPLC-ABTS^+•^ (910 ± 1.3 µmol Trolox^®^/g extract), and β-carotene (74.3 ± 0.8 µmol Trolox^®^/g extract).

## 1. Introduction

Various infectious diseases prevail in tropical countries because they have favorable environments for the propagation of microorganisms and biological vectors [1]. In Colombia, dengue is an infectious disease that affects a large part of the population (approximately 100,000 people every year) [2]. Dengue is transmitted by the vector *Aedes aegypti* [3], and to a lesser degree, *A. albopictus*; its symptoms are vomiting, fever, headache, and, in more serious conditions, bleeding. Infectious diseases, including dengue, are accompanied by high oxidative stress, which occurs when there is a disturbance in the balance between reactive oxygen species (ROS) and the body’s antioxidant defense mechanisms [4].

The search for antioxidant compounds (or their mixtures), preferably of natural origin, can be one of the alternatives to alleviate not just the course of infectious diseases, e.g., dengue or coronavirus [5], but also diseases like hyperuricemia [6] and diabetes [7]. The genus *Scutellaria* (Lamiaceae) includes approximately 460 species distributed worldwide, mainly in temperate regions of Europe, North America, and East Asia [8]. *Scutellaria* plants have long been employed in Asian and European regions [9]; their leaves, stems, and roots have been used to make infusions and ointments to control the symptoms of respiratory diseases such as the flu, asthma, and pneumonia, among others. In Colombia, the Paéz indigenous people (Cauca, southern Colombia) refer to *Scutellaria* by the name “*Alegría*” (happiness), because the infusion of leaves is also used to calm anxiety, depression and symptoms of respiratory diseases [10]. Various studies [11,12] indicate that *Scutellaria* spp. hydroethanolic extracts possess a wide variety of bioactive secondary metabolites with several biological properties, including anti-inflammatory, anti-allergenic, antioxidant, antiviral, and antitumor activities [13].

The antioxidant [14] and anti-inflammatory [15] activities of *Scutellaria* spp. have been reported. However, the contribution of individual compounds to the biological activity of their extracts is still unknown. Researchers such as Dong et al. [9] and Lim et al. [16] isolated phenolic compounds from extracts of these plants using low-pressure-preparative chromatography (LPPC) and column chromatography with different elution solvents in order to determine their antioxidant, antiviral, and antitumor properties. Wogonoside [17], baicalein [18], baicalin [19], wogonin [20], and oroxylin A [21], characteristic compounds present in *Scutellaria* spp. plants, have antioxidant, antiviral, and antitumor properties; they modulate various biosynthetic pathways related to cancer cell proliferation.

**Figure 1:**
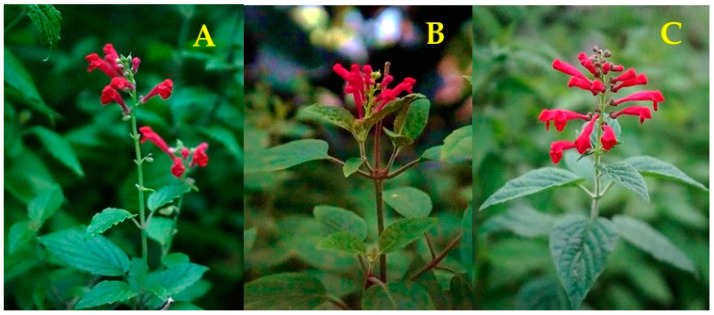
*Scutellaria* plants native to Colombia at their flowering stage: (**A**). *S. incarnata*; (**B**). *S. coccinea* (**C**). *S. ventenatii* × *S. incarnata*.

Colombia is a megabiodiverse country and has a very high variety of plants, with more than 2400 species, of which less than 15% have been phytochemically studied [22]. *Scutellaria* spp., native to Colombia, are easy to grow, spread rapidly, and are generally not attacked by predators. Despite this, there have been few studies on native *Scutellaria* spp. The objective of this work was to determine the chemical composition and antioxidant activity of the hydroethanolic extracts of dried plants of three *Scutellaria* species that grow in Colombia, namely, *S*. *incarnata*, *S*. *coccinea,* and *S*. *ventenatii* × *S*. *incarnata* (see Figure 1).

## 2. Results

### 2.1. Experimental Design and Extraction Yields

Analysis of variance (ANOVA) was used to determine the significance of the effects of time, temperature, and percentage of ethanol on the yield and antioxidant activity of the *Scutellaria* extracts obtained. Table 1 shows the results of the experimental design to obtain extracts from the studied *Scutellaria* spp., and Table 2 shows the conditions that allowed obtaining extracts with high extraction yields and high antioxidant activity.

### 2.2. Analysis with UHPLC/ESI-Q-Orbitrap-MS of Scutellaria spp. Extracts

**Figure 2:**
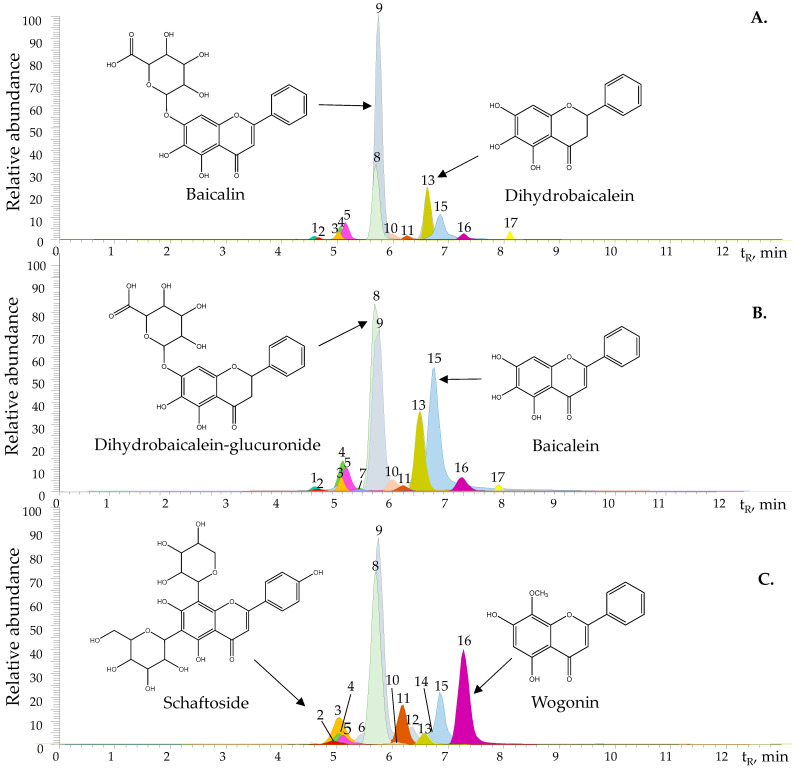
Extracted ion currents (EICs) obtained by UHPLC/ESI-Q-*Orbitrap*-MS in full scan mode of protonated molecules [M+H]^+^ from hydroalcoholic extracts of (**A**). *S. incarnata*, (**B**). *S. coccinea* and (**C**). *S. ventenatii* × *S. incarnata*. Table 3 shows peak identification.

The identification and quantification of secondary metabolites in the hydroalcoholic extracts of the three *Scutellaria* species studied was carried out by UHPLC/ESI-Q-Orbitrap-MS, operated in the dual mode of positive and negative ion acquisition. Compounds were detected in the total ionic current (TIC) using their exact masses, which were experimentally measured with Δppm < 2. The isotopic pattern of protonated or deprotonated molecules was consistent with that calculated by Thermo Xcalibur 3.1 software. The fragmentation pattern of [M+H]^+^ and [M−H]^−^ ions was studied using different energies (10, 20, 30, 40, 50, 60, or 70 eV) in a higher-energy collision dissociation cell (HCD). The collision energy values were chosen while keeping in mind the highest number of product ions registered without losing the [M+H]^+^ or [M−H]^−^ signals of phenolic substances. A total of 17 compounds were identified by comparing their mass spectra with those found in the literature and in spectral databases [23] and studying the fragmentation patterns, measuring the exact masses and isotopic ratio of protonated or deprotonated molecules. Eight identifications were confirmed using standard compounds (baicalin, baicalein, norwogonin, wogonin, wogonoside, apigenin-7-glucuronide, scutellarein, and scutellarin). Figure 2 shows the extracted ionic currents (EICs) of the molecules present in the TICs obtained in full scan acquisition mode. Table 3 and Table 4 show the identification of the substances and their amounts in the extracts, respectively.

### 2.3. Antioxidant Activity

The antioxidant activity, measured by a single technique, reflects the response of the sample corresponding only to the conditions and the mechanism involved in the assay employed. It is important to use several methods based on different action modes, e.g., on the mechanism by donation of protons (ORAC and β-carotene) or by donation of electrons (ABTS^+^) [30]. Table 5 shows the results obtained for each extract studied, for the individual standard substances, and for quercetin, used as a control compound.

## 3. Discussion

### 3.1. Evaluation of Experimental Variables

Analysis of variance (ANOVA, Table 1) was used to determine the significance of the effects of time, temperature, and percentage of ethanol on the yield and antioxidant activity of the *Scutellaria* extracts obtained. All of the independent variables had an effect on the antioxidant activity measured by the online HPLC-ABTS^+^ method. However, the extraction time did not induce changes in the antioxidant activity of the *S*. *incarnata* extract. Temperature had a higher effect on the antioxidant activity of the *S*. *incarnata* and *S*. *coccinea* extracts, while the temperature–ethanol percentage interaction was the most important for the *S*. *ventenatii* × *S. incarnata* extract (Figures S1–S3). The extraction yield was not modified by the same variables in all cases. For the *S*. *incarnata* extract, the variable with the highest effect was temperature; for the *S*. *coccinea* extract, it was the ethanol percentage. The results indicate the importance of evaluating the factors of temperature, ethanol–water ratio, and time for the extracts of each plant studied. For example, temperature was the factor with the greatest significant effect on the extraction yield of *S*. *incarnata*.

It has been found [33] that excess ROS in the organism accompany viral diseases; therefore, in the experimental design for extract obtainment, the combined response of both high yield and high antioxidant activity was evaluated. Table 2 shows the variables that allowed higher yields and antioxidant activities to be obtained. For all of the extracts, it was observed that the effects may change in the combined response. For example, the *S*. *incarnata* extract yield did not rise with the increase in the percentage of ethanol–water or with time, but the antioxidant activity turned out to be higher, so their combination was significant (Figures S1C, S2C and S3C).

### 3.2. Quantification of Phenolic Compounds in Scutellaria spp. Extracts

Secondary metabolites play various functions in the plant, including defense against different types of viruses and pests, insect repellency, and participation in communication between plants to favor or limit their growth [34]. There are various studies [35,36] on the chemical composition of secondary metabolites of species of the *Scutellaria* genus, but no reports on native species of Colombia have been found thus far. In this work, the chemical composition of the hydroethanolic extracts of the species *S*. *incarnata*, *S*. *coccinea*, and *S*. *ventenatii* × *S*. *incarnata*, native to Colombia, were studied using liquid chromatography coupled with high-resolution mass spectrometry (UHPLC/ESI-Q-Orbitrap-MS, Thermo Scientific, Waltham, MA, USA, and, Bremen, Germany).

Flavones were more abundant in the extracts, followed by flavanones, flavonols, and derivatives of hydroxycinnamic acids. The EICs of the protonated molecules of compounds present in the three *Scutellaria* spp. extracts studied are shown in Figure 2. The fragmentation patterns in mass spectra of some tentatively identified compounds are discussed below.

Dihydrobaicalein-glucuronide and its aglycone, dihydrobaicalein, have previously been detected at low concentrations in other *Scutellaria* species [24,25,27]. In this investigation, their fragmentation patterns were studied (Figures S4 and S5). The [M+H]^+^ protonated molecule of dihydrobaicalein-glucuronide at *m*/*z* 449.10783 fragments to give rise to the product ion at *m*/*z* 273.07575 by breakage of the glycosidic bond [M+H−C_6_H_8_O_6_]^+^. This ion decays, generating fragments [(M+H)−C_6_H_8_O_6_−H_2_O]^+^ at *m*/*z* 255.06497 and [(M+H) −C_6_H_8_O_6_−C_8_H_8_]^+^ at *m*/*z* 169.01292 due to the loss of water and the retro Diels–Alder reaction suffered by the aglycone, respectively. The latter process has been reported as characteristic of flavanones [37]. The product ion at *m*/*z* 131.04906, C_9_H_7_O, is generated by the loss of ring C of the aglycone, e.g., [(M+H)−C_6_H_8_O_6_−C_6_H_6_O_4_]^+^.

Apigenin-C-glucoside-C-arabinoside (schaftoside) was reported by Liu et al. [24] and Han et al. [25] in other *Scutellaria* species. In its (ESI/MS) mass spectrum, from the deprotonated molecule [M−H]^−^ at *m*/*z* 563.13953, losses of 60, 90, and 120 units are observed, which generate fragments at *m*/*z* 503.12198, 473.10934, and 443.09860, respectively. According to Becchi and Fraisse [38] and Wu et al. [39], these are typical ions produced by C-glycosidic bond cleavages. The ion at *m*/*z* 413.08817 is generated by the cleavage of the hexose [(M−H)−C_5_H_10_O_5_]^−^, which does not involve the rupture of the ^1′^C–^6^C bond between the aglycone and the glycoside (Figure S6). The product ion at *m*/*z* 383.07773 corresponds to the successive loss of 60 (−C_2_H_4_O_2_) and 120 (−C_4_H_8_O_4_) units in the sugars; the product ion at *m*/*z* 353.06668 is similarly formed by the successive losses of 60 and 150 units (−C_5_H_10_O_5_).

Umbelliferone-hexoside-pentoside, to the best of our knowledge, was found for the first time in *Scutellaria* spp. extracts. The ion [M+H]^+^ appears in the mass spectrum at *m*/*z* 471.14970 and corresponds to the elemental formula of this substance, C_21_H_27_O_12_. The [M+H]^+^ ion dissociates, generating the product ion [(M+H)−2H_2_O]^+^ at *m*/*z* 435.12823 by loss of two water molecules. The elimination of a molecule of rhamnose [(M+H)−C_6_H_10_O_4_]^+^ generates the product ion at *m*/*z* 325.09137, which decays, eliminating a glucose to form the fragment [(M+H)−C_6_H_10_O_4_−C_6_H_10_O_5_]+ at *m*/*z* 163.03882. In turn, this ion loses water, forming the product ion [(M+H)−C_6_H_10_O_4_−C_6_H_10_O_5_−H_2_O]^+^ at *m*/*z* 145.07590. The protonated [M+H]^+^ molecule of umbelliferone-hexoside-pentoside undergoes the loss of a glucose molecule [(M+H)−C_6_H_10_O_5_]^+^ to generate the product ion at *m*/*z* 309.09671 (Figure S7). This type of loss has been reported by Li and Claeys [40] and Ma et al. [41] for glycosylated flavonoids.

A compound (t_R_ = 8.55 min), corresponding to a deprotonated [M−H]^−^ molecule at *m*/*z* 329.17474 (C_20_H_26_O_4_) was not identified at a confirmatory level. The deprotonated molecule [M−H]^−^ undergoes the loss of a methyl radical with formation of the product [(M−H) −CH_3_]^−•^ at *m*/*z* 314.15262 (100%). The product ions at *m*/*z* 285.11313 (0.2%) and *m*/*z* 271.09641 (0.3%) correspond to the losses of the propane (C_3_H_8_) and butane (C_4_H_10_) molecules, respectively (Figure S8). In extracts of *Scutellaria* spp. diterpenes have been identified by LC/ESI-/MS [42,43,44] that, in their mass spectra, present product ions of the losses of alkyl radicals CH_3_^•^, C_3_H_7_^•^, C_5_H_11_^•^; however, the deprotonated [M−H]^−^ molecule at *m*/*z* 329.17474 in this study presents a fragmentation pattern slightly different from those of the diterpenes reported in *Scutellaria* spp. [42,43,44], since from the [M−H]^−^ ion, losses were observed not only of radicals, but also of molecules such as propane and butane.

Compound quantification was performed with the external standardization technique using [M+H]^+^ or [M−H]^−^ in the selected ion monitoring (SIM) mode. In the case of ions extracted from the TIC for which a reference substance was not available, quantification was performed using the calibration curve obtained for a structurally similar substance.

In the three extracts, baicalin was the major compound (Table 4). This coincides with what was found for *S*. *baicalensis* [9,45] and *S*. *lateriflora* [46]. Baicalin has been found in many species of this genus [35] and has been the subject of much interest in different biological studies. It has demonstrated its effectiveness in inhibiting HIV infection in vitro [47], reducing the oxidative stress produced by the cytotoxicity generated by H_2_O_2_ in HK-2 cells [48], inducing and enhancing apoptosis in HT-29 cells in a dose-dependent manner [49], and inhibiting the replication of dengue virus such as dengue serotype 2 [50]. The flavanone dihydrobaicalein-glucuronide was the second most-abundant compound in the extracts studied. However, no reports on its biological properties have been found.

The chemical compositions of the extracts of the three *Scutellaria* species were very similar. However, the amount and ratio of the compounds present in the extracts differed. This permitted distinguishing each one of the species studied. In all of the extracts, the glycosylated compounds were found together with their respective aglycones. Their extraction is explained by the type of solvent used (ethanol–water), which made it possible to isolate both very polar (glycosides) and moderately polar (aglycones) molecules and compounds with several −OH groups in their structures [51,52].

### 3.3. Antioxidant Activity of Scutellaria spp. Extracts

Table 5 presents the antioxidant activity values obtained with the ORAC, ABTS^+•^, online HPLC-ABTS^+•^, and β-carotene discoloration assays of hydroalcoholic extracts of *Scutellaria* spp. Results from different antioxidant assays were analyzed by ANOVA followed by Tukey’s post hoc test in order to establish significant differences (*p* < 0.05) between samples. The *S*. *coccinea* extract had the highest antioxidant activity in all the trials; this may be due to the amounts of baicalin and dihydrobaicalein-glucuronide (Section 2.2), which are appreciated in the HPLC-ABTS^+•^ profile (Figure S9). This agrees with what was reported by Lee et al. [53] for *S*. *baicalensis* extracts. The amounts of these compounds were higher in the *S*. *incarnata* extract (287.127 ± 0.005 mg/g and 140.18 ± 0.07 mg/g for baicalin and dihydrobaicalein-glucuronide, respectively). The even higher antioxidant activity of the *S*. *coccinea* extract may be due to the presence of isoliquiritin, which was found only in this extract. Isoliquiritin acts as an “activator” of superoxide dismutase and catalase enzymes [54,55] to control diseases such as cancer and cerebral ischemia.

No extract surpassed the antioxidant activity value of quercetin in any trial. However, when studying the standard compounds individually, it was found that scutellarein and apigenin presented a response very similar to that of quercetin in all techniques employed. This indicates that some compounds in the *Scutellaria* spp. extracts can act antagonistically; that is, they do not increase the antioxidant activity of the mixture but decrease it.

Using the online HPLC-ABTS^+•^ method, a relationship was established between the amount of compound present in each extract and its antioxidant activity. The higher the amount of dihydrobaicalein-glucuronide present in the extracts, the higher the measured antioxidant activity (Table S2). When the individual contributions of the compounds appearing in the chromatographic profile were added, the *S*. *incarnata* extract (140.18 ± 0.07 mg/g extract; 497 µmol Trolox^®^/g extract) had the highest sum, followed by *S*. *coccinea* (51.20 ± 0.02 mg/g extract; 382 µmol Trolox^®^/g extract) and *S*. *ventenatii* × *S*. *incarnata* (44.89 ± 0.06 mg/g extract; 163 µmol Trolox^®^/g extract). This indicates that, apart from the antagonistic interactions mentioned above, the antioxidant response was dose dependent. This coincides with what was reported by Seo et al. [28] for the methanolic extracts of *S*. *baicalensis*, analyzed by online HPLC-ABTS^+•^.

The antioxidant activity of phenolic compounds depends on their structure. The degree of glycosylation, as well as the position and number of hydroxyl groups, contribute to the antioxidant response related to the donation of hydrogen atoms or electrons to form more stable intermediates [56]. Some studies on the structure–activity (SAR) of flavonoids have shown that the antioxidant potential increases with the presence of (1) −OH groups on ring B; (2) −OH groups on ring A and a 4-oxo function on ring C; and (3) a double bond at positions 2 and 3 of ring C [57].

In the present study, it was observed that the flavanone dihydrobaicalein-glucuronide, a molecule without a double bond in positions 2 and 3 of the C ring, was the main contributor to the antioxidant response of *Scutellaria* spp. extracts (163 to 497 µmol Trolox^®^/g extract, Table S2). Baicalin, with a double bond in the C ring (position 2–3) and being the most abundant compound in the extracts, was the second most responsible for the antioxidant activity (24 to 137 µmol Trolox^®^/g extract). Cotelle [58] attributed this to the absence of −OH groups in the B ring of the molecule, which can “weaken” the action of the double bond in the central ring. However, Ishimoto et al. [59] discovered that although an increase in hydroxyl groups in the B ring of a flavonol has been related to its antioxidant activity, as measured by the ORAC method, kaempferol (with a hydroxyl group on the B ring) has antioxidant activity 1.5 times higher than that of quercetin and myricetin (with two and three hydroxyl groups on their respective B rings).

Among the standard substances evaluated individually (Table 5), the aglycones presented higher antioxidant activity with respect to their glycosylated derivatives measured by all the techniques used. The results agree with those obtained by Xie et al. [60] and Plumb et al. [61]. However, several studies [62,63,64] have shown that, compared to their aglycones, glycosides can improve compound stability, increase solubility in water, and reduce toxic effects, although they possess lower antioxidant activity. When ingested, the aglycones are degraded and lose their activity, but the glycosylated compounds can increase their activity due to the breaking of the *O*-glycosidic bond. Since the studied extracts were rich in glycosylated compounds, they could be used eventually in a phytotherapeutic product with high antioxidant value.

Bearing in mind that a 1 ha plantation of *Scutellaria* spp. contains approximately 50,000 plants, which can be harvested four times a year, ca. a total of 250 kg of plant material could be obtained per hectare per year. Therefore, the annual quantity of dry extract, considering a percentage of humidity of the material of ca. 80%, would be 13.5 kg, for a yield of 27%, with high amounts of baicalin (ca. 3.7 kg/ha/year) and dihydrobaicalein-glucuronide (ca. 1.8 kg/ha/year), substances of commercial interest due to their special biological activity.

## 4. Materials and Methods

### 4.1. Reagents

2,2′-Azino-bis-83-ethylbenzothiazolin-6-sulfonic acid (ABTS) (≥98%), gallic (≥97%) and linoleic (≥99%) acids, β-carotene (≥ 93%), chloroform (≥99%), baicalein (≥95%), apigenin (≥95%), and Trolox^®^ (≥97%) were purchased from Sigma-Aldrich (St. Louis, MO, USA). Salvigenin (≥98%), scutellarin (≥98%), scutellarein (≥98%), baicalin (≥98%), apigenin-7-glucuronide (≥98%), wogonin (≥98%), wogonoside (≥98%), eriodictyol-7-glucoside (≥98%), and norwogonine (≥98%) were obtained from Chemfaces (Wuhan, China). Galangin (≥95%), kaempferol-3-*O*-rutinoside (≥98%), and vitexin (≥98%) were purchased from Phytolab GmbH (Vestenbergsgreuth, Bavaria, Germany). HPLC-grade acetonitrile, HPLC-grade formic acid (FA), isopropanol (98%), ammonium formate (AF) (≥99%), LC/MS-grade methanol, and potassium persulfate (≥98%) were obtained from Merck (Darmstadt, Germany). Type I water was obtained from a Millipore Direct-QTM purification system.

### 4.2. Scutellaria Plants

The *Scutellaria* plant material was provided by the Colombian state through the No. 270 contract for access to genetic resources and derived products between the Ministry of Environment and Sustainable Development and the Industrial University of Santander. Leaves and stems of *S*. *incarnata* (voucher number UIS219783), *S*. *coccinea* (voucher number UIS219784), and *S*. *ventenatii* × *S*. *incarnata* (hybrid, voucher number UIS219785) were collected during flowering in experimental plots of the Pilot Agroindustrial Complex of the National Research Center for the Agro-industrialization of Tropical Aromatic and Medicinal Plant Species, CENIVAM (Bucaramanga, Santander, Colombia), in April 2021.

### 4.3. Experimental Design and Extraction

For the three *Scutellaria* species, a 2^3^ experimental design with a central point was developed in order to determine the best extraction conditions. An ultrasound-assisted solvent extraction (SE) technique was used; each point was performed in triplicate, while the central point was performed in quintuplicate. The modification factors were temperature (A), ethanol–water (solvent) ratio (B), and time (C). The combined response was determined by normalizing the yield and antioxidant activity data and summing their values. The data were processed with Statgraphics Centurion XVI software, version 16.1. For the extraction, dried and ground plants (1 g) were used, which were placed in a 100 mL amber bottle, to which an ethanol–water solution (20 mL) was added. The mixture was subjected to ultrasonication (35 kHz, Elmasonic S15H, Singen, Germany) and filtered (Wattman No. 1 paper), and the residue was extracted once more with ethanol–water (10 mL). The extracts were vacuum rotoevaporated at 50 °C in Heidolph equipment (Hei-VAP, Advantage HL, Chicago, IL, USA), dried in a VirTis AdVantage Plus tray lyophilizer (SP Scientific, Gardiner, NY, USA) and stored at 4 °C in the absence of light.

### 4.4. Identification and Quantification by LC/MS of Compounds in Scutellaria spp. Extracts

Samples were weighed (0.5 mg) and dissolved in mobile phase (1 mL) 50:50 methanol–water (0.1% formic acid and 5 mM ammonium formate) to a final concentration of 500 mg/L.

Phenolic compounds were analyzed on a VanquishTM ultra-high performance liquid chromatograph (UHPLC) (Thermo Scientific, Waltham, MA, USA), equipped with a thermostatically controlled column compartment (40 °C). Chromatographic separation was performed on a 50 mm L × 2.1 mm I.D. Zorbax Eclipse XDB C18 column, 1.8 µm particle size (Sigma-Aldrich, St. Louis, MO, USA). The flow rate of the mobile phase containing (A) water (0.1% FA + 5 mM AF) and (B) MeOH (0.1% FA + 5 mM AF) was 300 μL/min. The initial gradient condition was A-100%, which linearly changed to B-100% in 8 min, held for 4 min, returned to A-100% in 1 min, and remained so for 3 min. The injection volume was 2 μL. The UHPLC was connected to a Q-Exactive Plus Orbitrap mass spectrometer (Thermo Scientific, Bremen, Germany) with a heated electrospray ionization (HESI-II) source. The capillary voltage was 3.5 kV. The nebulizer temperature was set at 350 °C and the capillary temperature at 320 °C. The sheath gas and auxiliary gas (N_2_) were set to 40 and 10 arbitrary units, respectively. Nitrogen (>99% purity) was obtained from a generator (NM32LA, Peak Scientific, Glasgow, Scotland, UK). The ions injected into the HCD cell, coming from the C-trap, were fragmented, using normalized collision energies in steps from 10 to 70 eV. Data were acquired and analyzed using Thermo Scientific™ Dionex™ Chromatography Data System (CDS) software, version 7.2 and Thermo Xcalibur 3.1 software (Thermo Scientific, CA, USA). Identification of compounds was carried out by comparing their retention times (t_R_), exact ion masses, isotopic ratios, and fragmentation patterns with those of standard substances and databases [65,66].

### 4.5. In Vitro Antioxidant Activity of Scutellaria spp. Extracts

#### 4.5.1. ABTS^+•^ Cation-Radical Discoloration Technique

The ABTS^+•^ cation-radical bleaching assay was carried out according to Re et al. [67], with some modifications. The test was performed in a Modulus^®^ II microplate reader (Turner Biosystems Inc., Sunnyvale, CA, USA). ABTS (7 mM in acetate buffer) and PDS (2.45 mM) were mixed with sonication (30 min) and stored at 4 °C (24 h) in the absence of light. ABTS^+•^ (900 μL) was taken and diluted in acetate buffer until an absorbance of 0.71 ± 0.02 at λ = 750 nm was obtained. Each extract (2 mg) was dissolved in methanol (2 mL) and diluted in acetate buffer (20 mM, pH 4.5). The diluted extract (10 μL) and ABTS^+•^ solution (190 μL) were deposited in each well of the plate, and the absorbance was measured for 60 min.

#### 4.5.2. Online HPLC-ABTS ^+•^ Cation–Radical Decoloration Technique

The extract was analyzed in a high-efficiency liquid chromatograph, HPLC 1200 Infinity (Agilent Technologies, AT, Palo Alto, CA, USA), coupled to a Pinnacle PCX post-column derivatizer (Pickering Laboratories, Mountain View, CA, USA) in order to determine its ability to decolorize the cation–radical ABTS^+•^. Chromatographic analysis was performed using the method established by Sierra et al. [32] in a liquid chromatograph with a GEMINI C_18_ chromatographic column of 100 mm length × 4.6 mm I.D. × 5 μm particle size (Phenomenex, Torrance, CA, USA), AT G1315D diode array detector, AT G1311C quaternary pump, and AT G1329B autosampler. Ten microliters of each extract (10 g/L) were injected. The column temperature was maintained at 25 °C. An aqueous solution of FA at 0.5% (*v*/*v*) (A) and HPLC grade acetonitrile (B) were used as the mobile phase, with a flow of 1 mL/min and a programmed elution gradient of 98% A (0 min), 88% A (15 min), 88% A (15–23 min), 60% A (46 min), 10% A (71 min), 10% A (71–75 min), 98% A (80 min), and 98% A (80–85). The detector was set to the following wavelengths: λ = 245, 270, 290, and 515 nm.

The cation–radical ABTS ^+•^ was generated by the oxidation of ABTS with PDS in a 2.9:1 molar ratio. The ABTS^+•^ stock solution was prepared by direct contact of ABTS (110 mL, 2 mM, in Type I water) and PDS (0.7 mM). The cation–radical concentration in the reactor was 79 μM, which allowed for achieving an absorbance (0.71 ± 0.02) equal to that established in the cation–radical decolorization assay ABTS^+•^ carried out in the microplate reader (see Section 4.5.1). Data were processed with AT ChemStation software, Version B.0.03-SR1. The identification of the compounds was carried out by comparing their retention times, t_R_, and UV-Vis spectra with those of standard compounds.

#### 4.5.3. β-Carotene Discoloration Assay

The antioxidant activity was evaluated following the methodology described by Panovska et al. [68], with some modifications. The extracts (2 mg) were dissolved in methanol (2 mL) and diluted. From each solution, 10 µL were taken, deposited in a microplate, and calibrated with 190 µL of a β-carotene emulsion, which was prepared by weighing the β-carotene (2 mg) and dissolving it in chloroform (200 µL). Twelve microliters of this solution were taken and placed in a conical container with Tween^®^ 40 (100 mg) and linoleic acid (10 mg); the mixture was shaken at 3000 rpm for 30 s. The chloroform was evaporated under a nitrogen flow (3 min), Type I water (1 mL) was added, and it was stirred at 3000 rpm for 1 min. Subsequently, a 1% PDS solution (3 mL) was added as a co-oxidizing agent, and it was brought up to 25 mL with Type I water. The absorbance reading was performed at a wavelength of λ = 450 nm at 45 °C in a 96-well ModulusTM II Microplate Multimode Reader (Turner Biosystems Inc., Sunnyvale, CA, USA)

#### 4.5.4. Measurement of Oxygen Radical Absorbance Capacity

The oxygen radical absorbance capacity (ORAC) was determined according to Huang et al. [69], with some modifications. A Varioskan LUX VL0000D0 spectrophotometer (Thermo Scientific, Republic of Singapore) equipped with black 200 µL 96-well poly(styrene) microplates was used under the fluorescence module. The extract (1 mg) was dissolved in methanol (1 mL) and diluted in phosphate buffer (75 mM, pH 7.4). The diluted extract (25 μL) and fluorescein solution (150 μL, 0.0838 mM in phosphate buffer) were deposited in each well of the plate. The mixture was incubated at 37 °C (20 min), and then a solution of 2,2′-azobis(2-amidinopropane) dihydrochloride (AAPH, 25 µL, 78 mM, in phosphate buffer) was added. Antioxidant protection was determined by the difference between the area under the fluorescence curve (AUC) and that of each sample and the blank (25 μL of phosphate buffer).

## 5. Conclusions

The experimental design carried out to extract phenolic compounds from three *Scutellaria* species native to Colombia allowed us to identify that for *S*. *incarnata*, a temperature of 50 °C, an ethanol–water percentage of 70%, and an extraction time of 5 min were the parameters that provided its highest extraction yield and antioxidant activity, while for *S*. *coccinea* and *S*. *ventenatii* × *S*. *incarnata,* these values were 50 °C, 70% and 40%, and 15 min, respectively. For the first time, the nonvolatile secondary metabolite composition of three *Scutellaria* species native to Colombia was determined, as well as the presence of umbelliferone-hexoside-pentoside in their extracts, a compound not found in other species of the genus. Dihydrobaicalein-glucuronide and baicalin were the major components of the *S*. *incarnata* (287.127 ± 0.005 mg/g and 140.18 ± 0.07 mg/g), *S*. *coccinea* (158.3 ± 0.34 mg/g and 51.20 ± 0.02 mg/g), and *S*. *ventenatii* × *S*. *incarnata* (186.87 ± 0.01 mg/g and 44.89 ± 0.06 mg/g) extracts. The highest antioxidant activity, measured by different techniques, was found for the *S*. *coccinea* extract (3828 ± 3.0 µmol Trolox^®^/g extract). The online HPLC-ABTS^+•^ method allowed the identification of dihydrobaicalein-glucuronide and baicalin as the compounds that mainly contribute to the total antioxidant activity of *Scutellaria* spp. extracts, with 70%, 60%, and 50% of the activity for *S*. *incarnata*, *S*. *coccinea*, and *S*. *ventenatii* × *S*. *incarnata*, respectively. The *Scutellaria* spp. studied could be used to obtain antioxidant extracts with potential use in the pharmaceutical sector and would also be good candidates for the isolation, by advanced fractionation techniques (prep/HPLC), of individual bioactive compounds as natural ingredients for different phytotherapeutic products.

## Figures and Tables

**Table 1:** Results of the analysis of variance used to evaluate the effect of the extraction conditions on the yield and antioxidant activity of hydroalcoholic extracts obtained from *S*. *incarnata*, *S*. *coccinea* and *S*. *ventenatii* × *S*. *incarnata* dried plants.

Species	*S. incarnata*				*S. coccinea*				*S. ventenatii* × *S. incarnata*			
Variable	Yield		AA		Yield		AA		Yield		AA	
Source	F	*p*	F	*p*	F	*p*	F	*p*	F	*p*	F	*p*
A: Temperature	20.59	0.001	61.26	0	4.22	0.064	24.65	0.001	17.61	0.001	368.69	0
B: Solvent	0.15	0.709	60.39	0	131.89	0.000	22.09	0.001	12.23	0.005	2589.6	0
C: Time	0.64	0.441	2.89	0.117	35.09	0.001	18.52	0.001	0.49	0.499	3300.1	0
AB	0.2	0.663	28.9	0.002	1.06	0.326	7.85	0.017	20.35	0.001	6680.8	0
AC	9.22	0.011	0.65	0.436	16.40	0.002	25.54	0.001	0.28	0.61	5.57	0.042
BC	4.31	0.062	30.72	0.001	5.00	0.050	8.10	0.016	15.61	0.002	67.84	0

AA—Antioxidant activity measured by the online HPLC-ABTS^+•^ technique. A *p* value < 0.05 indicates that the effect is significant with a 95% confidence level. F-test.

**Table 2:** Conditions for higher yield and antioxidant activity, measured by the online HPLC-ABTS^+•^ assay, of *Scutellaria* spp. extracts.

Species	Combined Response			Yield, % ± s (*n* = 3)	Online HPLC-ABTS^+•^, µmol Trolox^®^/g extract ± s (*n* = 3) *
	Temperature, °C	EtOH−H_2_O, %	Time, min		
*S. incarnata*	50	70	5	26.0 ± 0.6	870 ± 3.5
*S. coccinea*	50	70	15	22.1 ± 0.4	920 ± 4.9
*S. ventenatii* × *S. incarnata*	50	40	15	19.4 ± 0.2	500 ± 2.1

* Sum of µmol Trolox^®^/g extract contributions from HPLC profile.

**Table 3:** Exact masses obtained by UHPLC/ESI-Q-*Orbitrap*-MS of protonated [M+H]^+^ and deprotonated [M−H]^−^ molecules and product ions of phenolic compounds present in *Scutellaria* spp. extracts.

Peak N° Figure 2	Compound	Formula	Experimental Masses, *m*/*z* (I, %)	Δppm	HCD, eV	Product Ions	Formula	*m*/*z* (I, %)	Identification Criteria	Refs
1	Isocarthamidin-glucuronide	C_21_H_20_O_12_	[M+H]^+^ 465.13580 (2%)	0.45	20	[(M+H)−C_6_H_8_O_6_]^+^	C_15_H_13_O_6_	289.07043 (100%)	a,b	[24]
						[(M+H)−C_6_H_8_O_6_−C_8_H_8_O]^+^	C_8_H_8_O	169.01312 (15%)		
						[(M+H)−C_6_H_8_O_6_−H_2_O]^+^	C_15_H_11_O_5_	271.06006 (1%)		
						[(M+H)−C_6_H_8_O_6_−H_2_O−C_6_H_4_O_3_]^+^	C_9_H_7_O_2_	147.04401 (6%)		
2	Apigenin-*C*-glucoside-*C*-arabinoside (*Schaftoside*)	C_26_H_28_O_14_	[M−H]^−^ 563.14191 (75%)	1.13	30	[(M−H)−C_2_H_4_O_2_]^−^	C_24_H_23_O_12_	503.12198 (5%)	a,b	[25]
						[(M−H)−C_3_H_6_O_3_]^−^	C_23_H_21_O_11_	473.10934 (64%)		
						[(M−H)−C_4_H_8_O_4_]^−^	C_22_H_17_O_10_	443.09860 (70%)		
						[(M−H)−C_5_H_10_O_5_]^−^	C_21_H_17_O_9_	413.08817 (13%)		
						[(M−H)−C_2_H_4_O_2_−C_4_H_8_O_4_]^−^	C_20_H_15_O_8_	383.07773 (68%)		
						[(M-H)−C_2_H_4_O_2_−C_5_H_10_O_5_]^−^	C_19_H_13_O_7_	353.06668 (100%)		
3	Verbascoside	C_29_H_36_O_15_	[M−H]^−^ 623.19704 (7%)	1.13	30	[(M−H)−C_9_H_6_O_3_]^−^	C_20_H_29_O_12_	461.16672 (14%)	a,b	[26]
						[(M−H)−C_9_H_6_O_3_−C_6_H_10_O_4_]^−^	C_14_H_19_O_8_	315.10852 (3%)		
						[(M−H)−C_20_H_30_O_12_]^−^	C_9_H_5_O_3_	161.02365 (100%)		
4	Umbelliferone-hexoside-pentoside	C_21_H_27_O_12_	[M+H]^+^ 471.14954 (20%)	1.00	10	[(M+H)−_2_H_2_O]^+^	C_21_H_23_O_10_	435.12823 (5%)	a	-
						[(M+H)−C_6_H_10_O_4_]^+^	C_15_H_17_O_8_	325.09261 (100%)		
						[(M+H)−C_6_H_10_O_5_]^+^	C_15_H_17_O_7_	309.09671 (10%)		
						[(M+H)−C_6_H_10_O_4_−C_6_H_10_O_5_]^+^	C_9_H_7_O_3_	163.03836 (17%)		
						[(M+H)−C_6_H_10_O_4_−C_6_H_10_O_5_−H_2_O]^+^	C_9_H_5_O_2_	145.07590 (13%)		
5	Scutellarin	C_21_H_18_O_12_	[M+H]^+^ 463.09691 (40%)	0.42	10	[(M+H)−C_6_H_8_O_6_]^+^	C_15_H_11_O_6_	287.05537 (49%)	a,b,c	[24]
						[(M+H)−C_6_H_8_O_6_−C_6_H_4_O_3_]^+^	C_9_H_7_O_3_	163.03885 (9%)		
6	Apigenin-7-glucuronide	C_21_H_18_O_11_	[M+H]^+^ 447.09056 (12%)	0.54	20	[(M+H)−C_6_H_8_O_6_]^+^	C_15_H_11_O_5_	271.06033 (100%)	a,b,c	[24]
						[(M+H)−C_6_H_8_O_6_−C_8_H_6_]^+^	C_7_H_5_O_5_	169.01256 (1%)		
7	Isoliquiritin	C_21_H_22_O_9_	[M+H]^+^ 419.13366 (22%)	1.07	10	[(M+H)−C_6_H_10_O_5_]^+^	C_15_H_13_O_4_	257.08054 (100%)	a,b	[23]
						[(M+H)−C_6_H_10_O_5_−H_2_O]^+^	C_15_H_11_O_4_	239.06585 (7%)		
8	Dihydrobaicalein glucuronide	C_21_H_20_O_11_	[M+H]^+^ 449.10602 (3%)	0.44	10	[(M+H)−C_6_H_8_O_6_]^+^	C_15_H_11_O_5_	273.07538 (100%)	a,b	[24]
						[(M+H)−C_6_H_8_O_6_−C_8_H_8_]^+^	C_7_H_5_O_5_	169.01341 (82%)		
						[(M+H)−C_6_H_8_O_6_−C_6_H_6_O_4_]^+^	C_9_H_7_O	131.04932 (25%)		
9	Baicalin	C_21_H_18_O_11_	[M+H]^+^ 447.09056 (5%)	0.33	20	[(M+H)−C_6_H_8_O_6_]^+^	C_15_H_11_O_5_	271.06033 (100%)	a,b,c	[24]
						[(M+H)−C_6_H_8_O_6_−C_8_H_6_]^+^	C_7_H_5_O_5_	169.01256 (1%)		
10	Scutellarein	C_15_H_10_O_6_	[M+H]^+^ 287.05444 (100%)	0.57	60	[(M+H)−H_2_O]^+^	C_15_H_9_O_5_	269.04416 (16%)	a,b,c	[24]
						[(M+H)−H_2_O−CO]^+^	C_14_H_9_O_4_	241.04912 (8%)		
						[(M+H)−C_8_H_6_O]^+^	C_7_H_5_O_5_	169.01280 (24%)		
						[(M+H)−C_8_H_6_O−CO]^+^	C_6_H_5_O_4_	141.01810 (10%)		
						[(M+H)−C_8_H_6_O−CO−H_2_O]^+^	C_6_H_3_O_3_	123.00771 (56%)		
						[(M+H)−C_7_H_5_O_5_]^+^	C_8_H_7_O	119.04921 (32%)		
11	Wogonoside	C_22_H_20_O_11_	[M+H]^+^ 461.17773 (61%)	20	0.47	[(M+H)−C_6_H_8_O_6_]^+^	C_16_H_13_O_5_	285.07535 (100%)	a,b,c	[24]
						[(M+H)−C_6_H_8_O_6_−CH_3_]^+^	C_15_H_10_O_5_	270.05164 (2%)		
12	Norwogonin-glucuronide	C_21_H_18_O_11_	[M+H]^+^ 447.20953 (52%)	20	0.24	[(M+H)−C_6_H_8_O_6_]^+^	C_15_H_11_O_5_	271.06003 (100%)	a,b	[24]
						[(M+H)−C_6_H_8_O_6_−C_8_H_6_]^+^	C_7_H_5_O_5_	169.01256 (1%)		
13	Dihydrobaicalein	C_15_H_12_O_5_	[M+H]^+^ 273.07617 (28%)	40	0.41	[(M+H)−H_2_O]^+^	C_15_H_11_O_4_	255.06465 (2%)	a,b	[24]
						[(M+H)−C_2_H_2_O]^+^	C_13_H_11_O_4_	231.06482 (1%)		
						[(M+H)−C_8_H_8_]^+^	C_7_H_5_O_5_	169.01292 (100%)		
						[(M+H)−C_6_H_6_O_4_]^+^	C_9_H_7_O	131.04906 (34%)		
						[(M+H)−C_7_H_5_O_5_]^+^	C_8_H_7_	103.05447 (4%)		
14	Norwogonina	C_15_H_10_O_5_	[M+H]^+^ 271.05969 (100%)	60	0.31	[(M+H)−H_2_O]^+^	C_15_H_9_O_4_	253.04919 (2%)	a,b,c	[27]
						[(M+H)−H_2_O−CO]^+^	C_14_H_9_O_3_	225.05435 (4%)		
						[(M+H)−C_8_H_8_]^+^	C_7_H_5_O_5_	169.01305 (28%)		
						[(M+H)−C_8_H_8_−CO]^+^	C_6_H_5_O_4_	141.01811 (5%)		
						[(M+H)−C_8_H_8_−CO−H_2_O]^+^	C_6_H_3_O_3_	123.00773 (6%)		
						[(M+H)−C_7_H_5_O_5_]^+^	C_8_H_7_	103.04451 (16%)		
15	Baicalein	C_15_H_10_O_5_	[M+H]^+^ 271.05969 (100%)	60	0.41	[(M+H)−H_2_O]^+^	C_15_H_9_O_4_	253.04919 (1%)	a,b,c	[27]
						[(M+H)−H_2_O−CO]^+^	C_14_H_9_O_3_	225.05435 (7%)		
						[(M+H)−C_8_H_6_]^+^	C_7_H_5_O_5_	169.01305 (16%)		
						[(M+H)−C_8_H_6_−CO]^+^	C_6_H_5_O_4_	141.01811 (11%)		
						[(M+H)−C_8_H_6_−CO−H_2_O]^+^	C_6_H_3_O_3_	123.00773 (63%)		
						[(M+H)−C_7_H_4_O_5_]^+^	C_8_H_7_	103.04451 (10%)		
16	Wogonin	C_16_H_12_O_5_	[M+H]^+^ 285.07575 (8%)	50	0.49	[(M+H)−CH_3_]^+^	C_15_H_10_O_5_	270.05176 (100%)	a,b,c	[27]
						[(M+H)−CH_3_−CO]^+^	C_14_H_10_O_4_	242.05701 (1%)		
						[(M+H)−CH_3_−C_8_H_7_]^+^	C_7_H_4_O_5_	168.07658 (3%)		
						[(M+H)−CH_3_−C_7_H_4_O_5_]^+^	C_8_H_7_	103.05444 (2%)		
17	Phenolic diterpene	C_20_H_26_O_4_	[M-H]^−^ 329.17474 (15%)	40	0.92	[(M−H)−CH_3_]^−^	C_19_H_22_O_4_	314.15262 (100%)	a	-
						[(M−H)−_2_CH_3_]^−^	C_18_H_19_O_4_	299.12906 (0.1%)		
						[(M−H)−C_3_H_8_]^−^	C_17_H_17_O_4_	285.11313 (0.2%)		
						[(M−H)−C_4_H_10_]^−^	C_16_H_15_O_4_	271.09641 (0.3%)		

^a^ Tentative identification was based on exact mass and isotopic ratio measurements. ^b^ Tentative identification was based on data from the scientific literature on the secondary metabolites from *Scutellaria* spp. plants [24,25,26,27,28,29]. ^c^ Confirmatory identification was based on comparison of the t_R_ and mass spectra with those of the reference substances. HCD—higher energy collision dissociation cell.

**Table 4:** Amounts (mg/g) of phenolic compounds present in extracts obtained from three *Scutellaria* spp. grown in Colombia.

Compound	mg Compound/g Dry Extract, Value ± s (*n* = 3)		
	*S. incarnata*	*S. coccinea*	*S. ventenatii* × *S. incarnata*
Baicalein	16.37 ± 0.01	18.55 ± 0.04	12.80 ± 0.01
Baicalin	278.127 ± 0.005	158.3 ± 0.3	186.87 ± 0.01
Wogonin	0.159 ± 0.004	0.7 ± 0.3	14.883 ± 0.006
Wogonoside	1.25 ± 0.06	1.241 ± 0.007	11.84 ± 0.04
Scutellarein	2 ± 1	3.79 ± 0.01	0.60 ± 0.02
Scutellarin	33.77 ± 0.02	21.400 ± 0.007	20.09 ± 0.03
Norwogonin	<LOD	<LOD	4.24 ± 0.04
Dihydrobaicalein ^a^	7.295 ± 0.009	8.843 ± 0.006	2.21 ± 0.07
Dihydrobaicalein glucuronide ^b^	140.18 ± 0.07	51.20 ± 0.02	44.89 ± 0.06
Norwogonin-glucuronide ^c^	<LOD	<LOD	29.79 ± 0.04
Isocarthamidin-glucuronide ^d^	13.117 ± 0.005	11.419 ± 0.007	<LOD
Apigenin-*C*-glucoside-*C*-arabinoside (*Schaftoside*) ^e^	0.83 ± 0.02	0.84 ± 0.02	0.43 ± 0.03
Verbascoside ^f^	33.72 ± 0.04	32.17 ± 0.06	41.54 ± 0.03
Umbelliferone-hexoside-pentoside ^g^	0.423 ± 0.008	0.60 ± 0.05	0.395 ± 0.003
Apigenin-7-glucuronide ^b^	<LOD	<LOD	9.24 ± 0.03
Isoliquiritin ^h^	<LOD	1.56 ± 0.05	<LOD

^a^ Quantification in baicalein equivalents. ^b^ Quantification in baicalin equivalents. ^c^ Quantification in wogonoside equivalents. ^d^ Quantification in eriodyctiol-7-glucoside equivalents. ^e^ Quantification in vitexin equivalents. ^f^ Quantification in 1,3-dicafeoylquinic acid equivalents. ^g^ Quantification in scopoletin equivalents. ^h^ Quantification in rosmarinic acid equivalents. LOD: Limit of detection. Norwogonin (LOD = 0.96 mg/g), wogonoside (LOD = 0.05 mg/g), eriodyctiol-7-glucoside (LOD = 0.04 mg/g), baicalin (LOD = 0.80 mg/g), and scopoletin (LOD = 0.05 mg/g).

**Table 5:** In vitro antioxidant activity of standard compounds and of *Scutellaria* spp. extracts.

Extracts/Compounds	Antioxidant Activity Assay			
	ORAC, µmol Trolox^®^/g Extract ± s, (*n* = 3)	ABTS^+.^, µmol Trolox^®^/g Extract ± s, (*n* = 3)	Online HPLC-ABTS^+•^, µmol Trolox^®^/g Extract ± s, (*n* = 2) *	β-Carotene, µmol Trolox^®^/g Extract ± s, (*n* = 3)
*S. coccinea*	3830 ± 3 ^a^	750 ± 1 ^a^	931 ± 3 ^a^	74.3 ± 0.8 ^a^
*S. incarnata*	2510 ± 2 ^c^	680 ± 1 ^b^	844 ± 2 ^b^	70 ± 1 ^b^
*S. ventenatii* × *S. incarnata*	2900 ± 2 ^b^	420 ± 1 ^c^	503 ± 2 ^c^	34 ± 1 ^c^
Baicalein	7150 ± 1	11,920.3 ± 0.3	11,560 ± 1	−
Baicalin	4736.0 ± 0.9	2570 ± 1	3522.12 ± 0.06	−
Scutellarein	22,830 ± 1	11,090 ± 1	12,124.9 ± 0.3	−
Scutellarin	12,004.9 ± 0.8	1744.5 ± 0.7	640 ± 1	−
Wogonin	9060 ± 1	5503.4 ± 0.5	90 ± 3	−
Wogonoside	3710 ± 1	1120 ± 2	−	−
Apigenin	29,420 ± 3	4100 ± 1	−	−
Apigenin glucuronide	10,670 ± 3	520 ± 1	−	−
Norwogonin	6100 ± 3	6323.4 ± 0.6	−	−
Quercetin	25,691.2 ± 0.6	11,100 ± 1	9228.3 ± 0.4	100 ± 1
Quercetin [31,32]	35,550 ± 1	10,000 ± 1	13,900 ± 99	−

* Sum of µmol Trolox^®^/g extract contributed by each compound in the extract. − Antioxidant activity not determined for these compounds. ^a–c^ Statistically significant differences in antioxidant activity among different *Scutellaria* spp. plants in the same in vitro assay, evaluated by ANOVA followed by Tukey’s post hoc test (*p* < 0.05); different letters indicate difference between groups.

## Data Availability

The supporting data are found in the database of the CIBIMOL research group, Universidad Industrial de Santander, Bucaramanga, Colombia.

## Supplementary Materials

The following supporting information can be downloaded at: https://www.mdpi.com/article/10.3390/molecules28083474/s1, Figure S1. Standardized Pareto diagrams for A. extraction yield; B. antioxidant activity; and C. combined response in the hydroalcoholic extract of *S. incarnata*. Figure S2. Standardized Pareto diagrams for A. extraction yield; B. antioxidant activity; and C. combined response in the hydroalcoholic extract of *S*. *coccinea*. Figure S3. Standardized Pareto diagrams for A. extraction yield; B. antioxidant activity; and C. combined response in the hydroalcoholic extract of *S. ventenatii* × *S. incarnata*. Figure S4. Mass spectrum of dihydro-baicalein-glucuronide, [M+H]^+^ at *m*/*z* 449.10783 (C_21_H_20_O_11_), present in the hydroalcoholic extract of *S. incarnata*. HPLC/ESI-Q-Orbitrap-MS, HCD, 20 eV. Figure S5. Mass spectrum of dihydrobaicalein, [M+H]^+^ at *m*/*z* 273.07575 (C_15_H_12_O_5_), present in the hydroalcoholic extract of *S. incarnata*. HPLC/ESI-Q-Orbitrap-MS, HCD, 40 eV. Figure S6. Mass spectrum of apigenin-C-glucoside-C-arabinoside, [M−H]^−^ at *m*/*z* 563.14191 (C_26_H_28_O_14_), present in the hydroalcoholic extract of *S. incarnata*. HPLC/ESI-Q-Orbitrap-MS, HCD, 30 eV. Figure S7. Mass spectrum of umbelliferone-hexoside-pentoside, [M+H]^+^ at *m*/*z* 471.14954 (C_21_H_27_O_12_), present in the hydroalcoholic extract of *S. incarnata*. HPLC/ESI-Q-Orbitrap-MS, HCD, 10 eV. Figure S8. Mass spectrum of the unidentified compound, [M−H]^−^ at *m*/*z* 329.17587 (C_20_H_26_O_4_), present in the hydroalcoholic extract of *S. incarnata*. HPLC/ESI-Q-Orbitrap-MS, HCD, 50 eV. Figure S9. Chromatographic profiles obtained by online HPLC-ABTS^+•^ of the extracts of A. *S. incarnata*; B. *S. coccinea* and C. *S. ventenatii* × *S. incarnata*, before (DAD, λ = 290 nm, blue line) and after (MWD, λ = 734 nm, red line) their reaction with the cation-radical ABTS^+•^. 1. Isocarthamidin-glucuronide; 2. Verbascoside; 3. Scutellarin; 4. Baicalin; 5. Dyhydrobaicalein-glucuronide; 6. Dyhidrobaicalein-baicalein; 7. Baicalein. ISTD-gallic acid (10 mg/L). Table S1. Experimental design with a central point used for the extraction of the three species of *Scutellaria*. Table S2. In vitro antioxidant activity of the compounds present in the extracts of *Scutellaria* spp., measured by the online HPLC-ABTS^+•^ method. References [70,71,72,73] are cited in the Supplementary Materials.

## Sample Availability

Samples of the compounds are available from the authors.
