# Supplementary material for: Chemical Characterization and Determination of the Antioxidant Properties of Phenolic Compounds in Three Scutellaria sp. Plants Grown in Colombia
Source: Molecules. 2023 Apr 14;28(8):3474. doi: 10.3390/molecules28083474 (PMC10142030; doi:10.3390/molecules28083474)
Supplement: Supplementary file 1 [file molecules-28-03474-s001.zip › molecules-2296373-supplementary.pdf]

Chemical characterization and determination of the antioxidant properties of phenolic compounds in three *Scutellaria* sp. plants grown in Colombia

Table S1. Experimental design with a central point, used for the extraction of three species of the *Scutellaria* genus

Experiment N°[a]	A (Temperature °C)	B (Ethanol-water, %)	C (Time, min)	*S. incarnata*		*S. coccinea*		*S. ventenatii* × *S. incarnata*	
				Yield, %	AA, µmol Trolox®/gE	Yield, %	AA, µmol Trolox®/gE	Yield, %	AA, µmol Trolox®/gE
1	30	40	5	19.55	587.5	24.08	657.5	18.40	117.5
2	50	40	5	26.07	840.5	23.08	625.0	17.62	259.0
3	30	70	5	21.96	912.5	19.85	893.5	16.91	345.0
4	50	70	5	26.03	867.5	15.76	907.5	19.32	380.5
5	30	40	15	24.12	697.0	25.06	516.0	18.73	47.50
6	50	40	15	23.30	849.0	25.19	1165.0	19.38	503.5
7	30	70	15	20.54	775.5	20.48	809.5	16.66	904.5
8	50	70	15	23.44	839.0	22.02	916.5	18.13	358.0
9	40	50	70	22.93	759.0	22.12	640.0	18.43	827.0

[a] Experiments were made in triplicate, and in quintuplicate for the central point.
AA: Antioxidant activity.
gE: gram per extract.

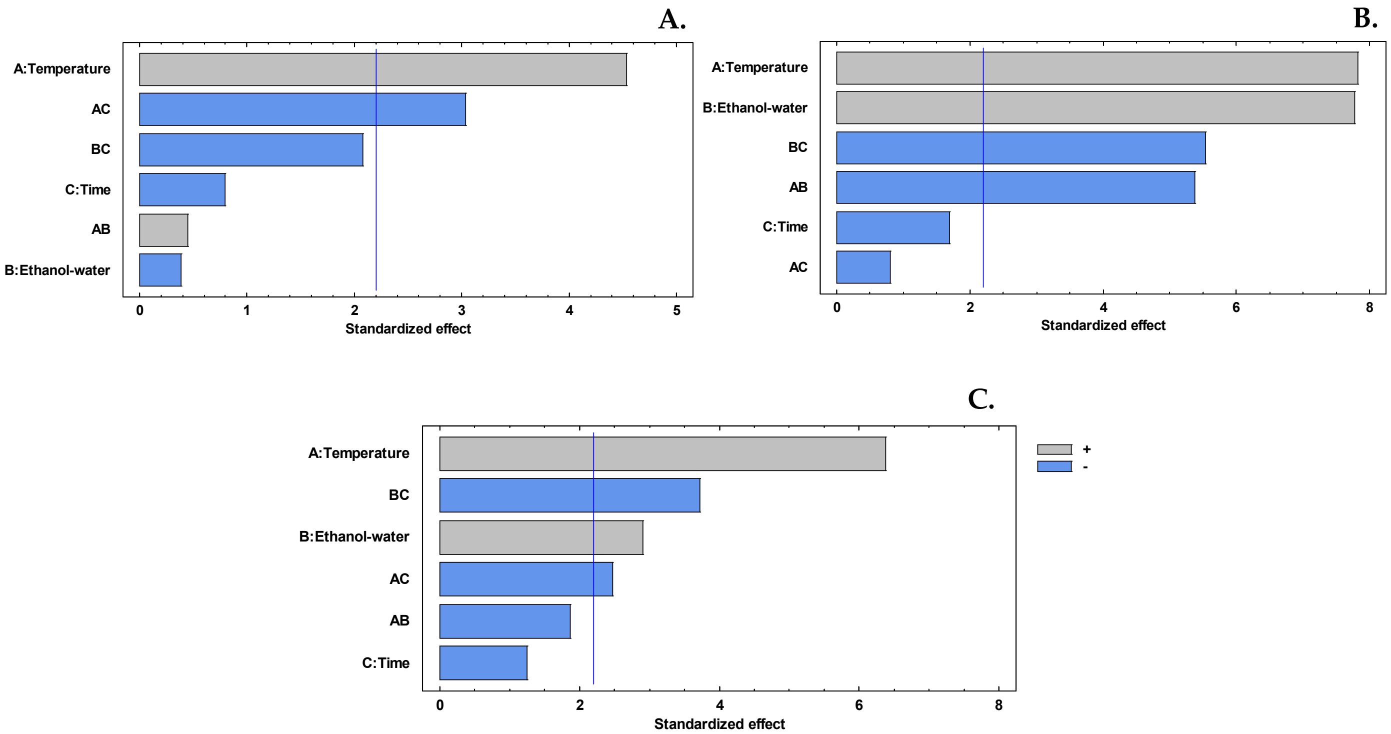

Figure S1. Standardized Pareto diagrams for: **A.** Extraction yield; **B.** The antioxidant activity and **C.** The combined response in the hydroalcoholic extract of *S. incarnata*.

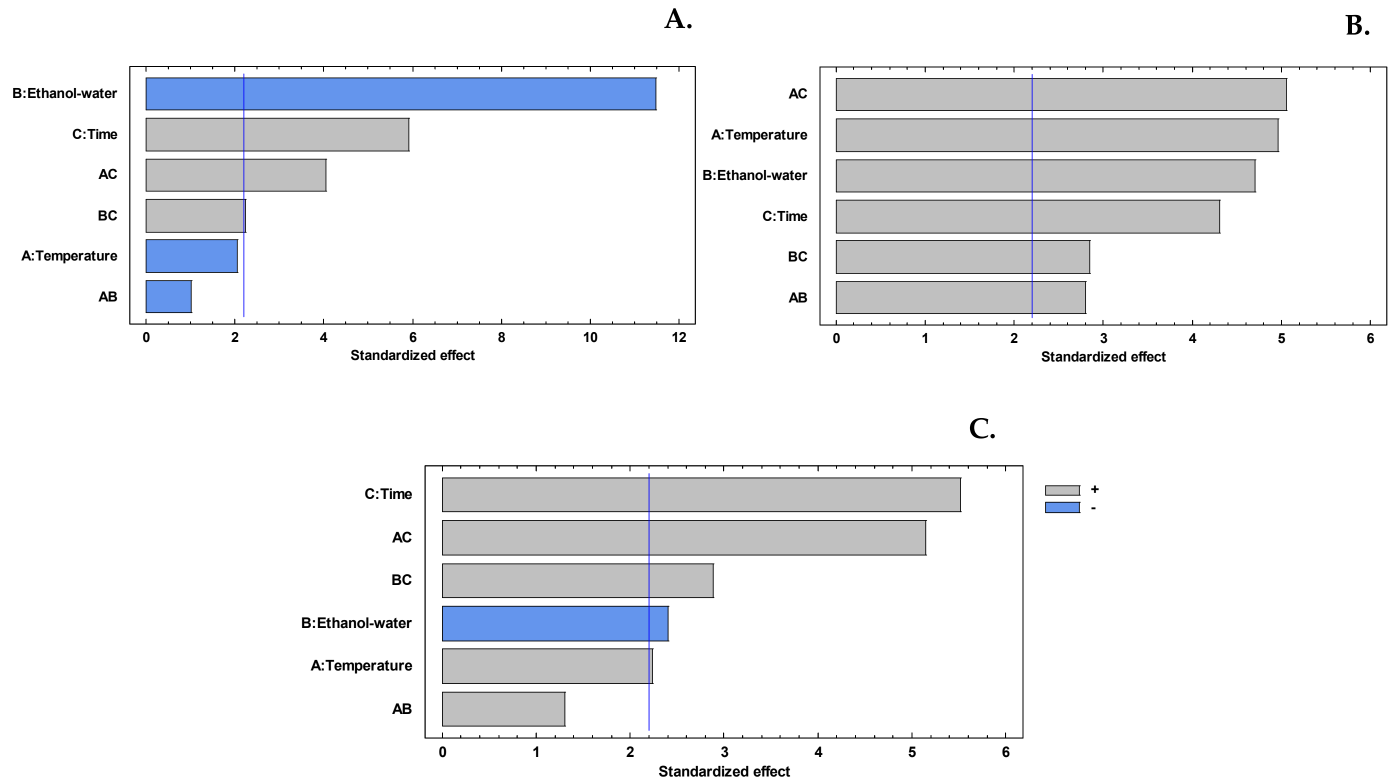

Figure S2. Standardized Pareto diagrams for: **A.** Extraction yield; **B.** The antioxidant activity and **C.** The combined response in the hydroalcoholic extract of *S. coccinea.*

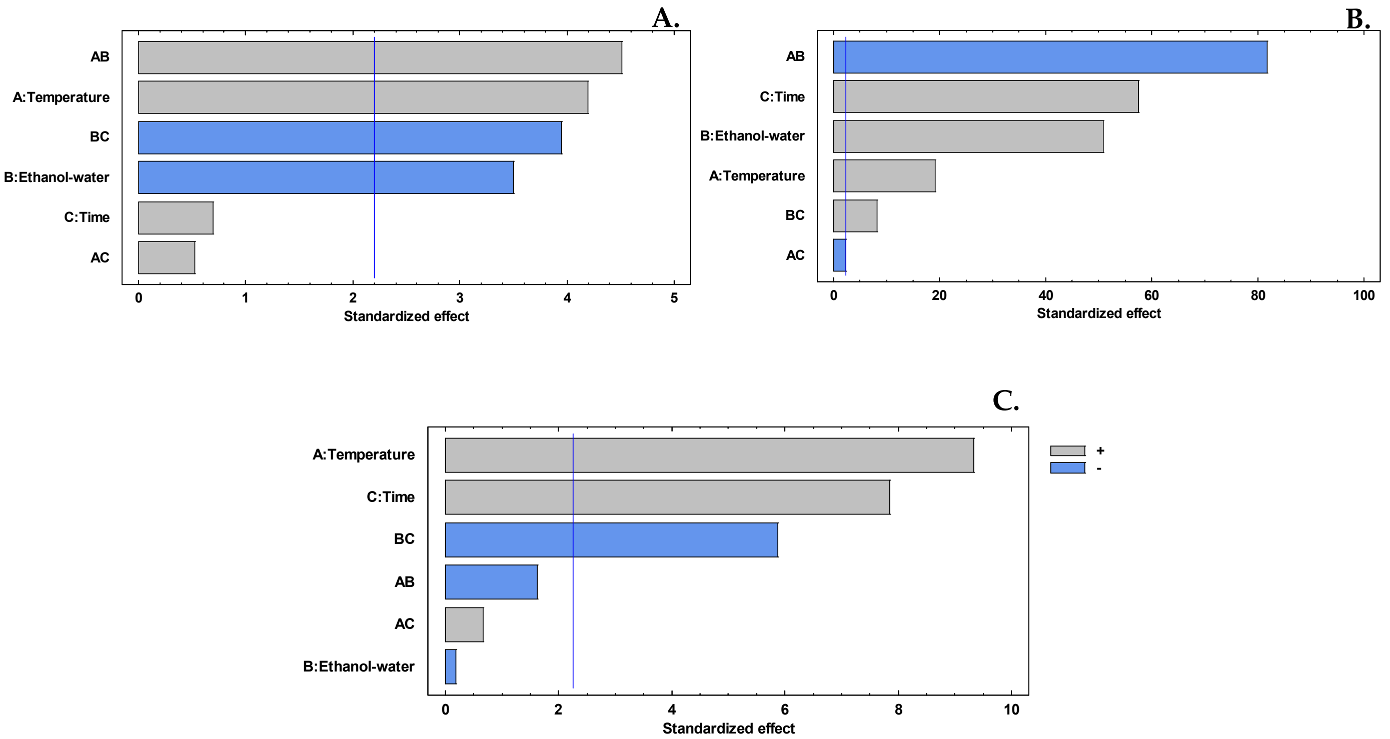

Figure S3. Standardized Pareto diagrams for **A.** Extraction yield; **B.** The antioxidant activity and **C.** The combined response in the hydroalcoholic extract of *S. ventenatii* × *S. incarnata.*

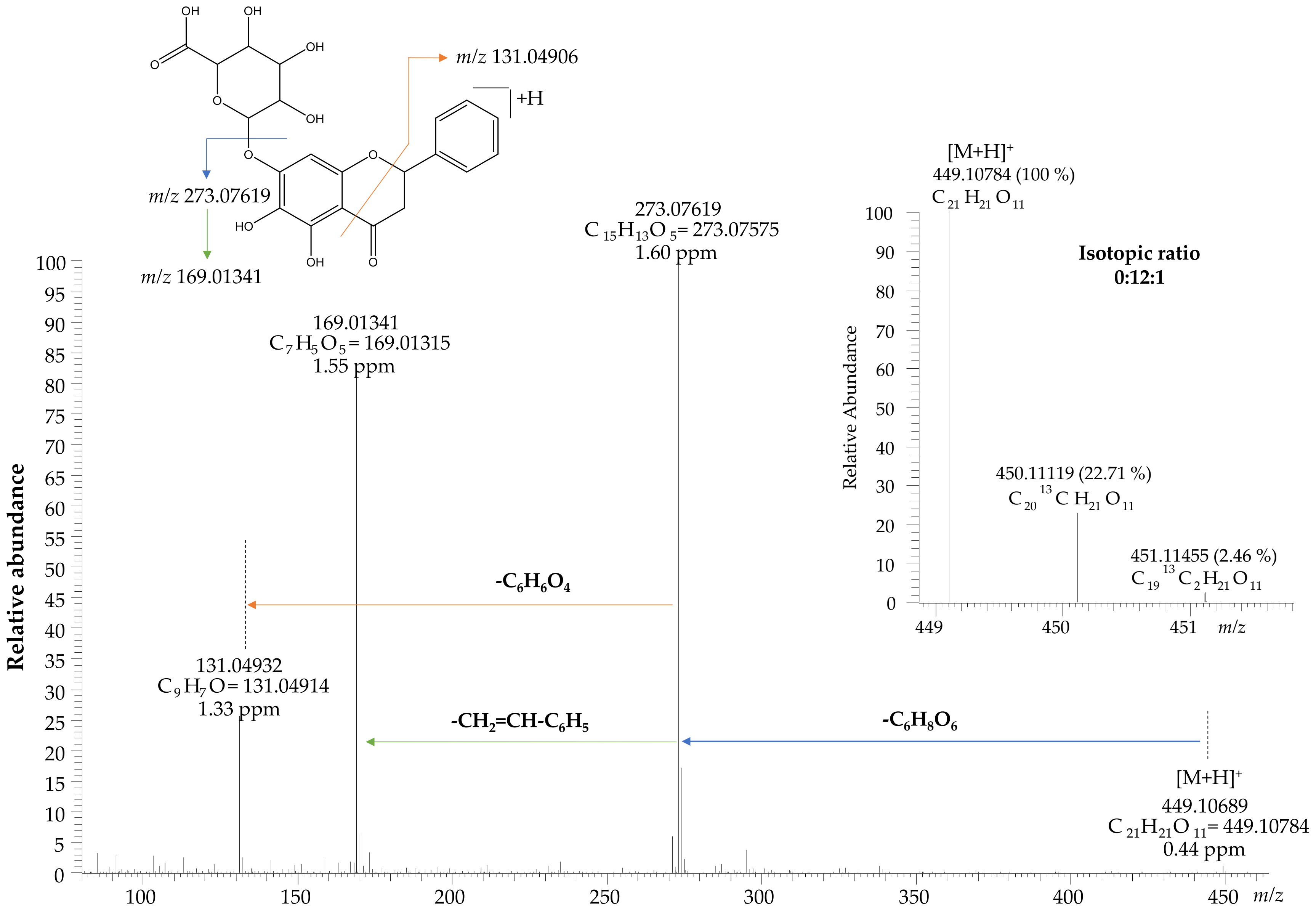

Figure S4. Mass spectrum of dihydrobaicalein-glucuronide, $[M+H]^+$ at *m/z* 449.10783 ($C_{21}H_{20}O_{11}$), present in the hydroalcoholic extract of *S. incarnata*. UHPLC/ESI-Q-Orbitrap-MS, HCD, 20 eV.

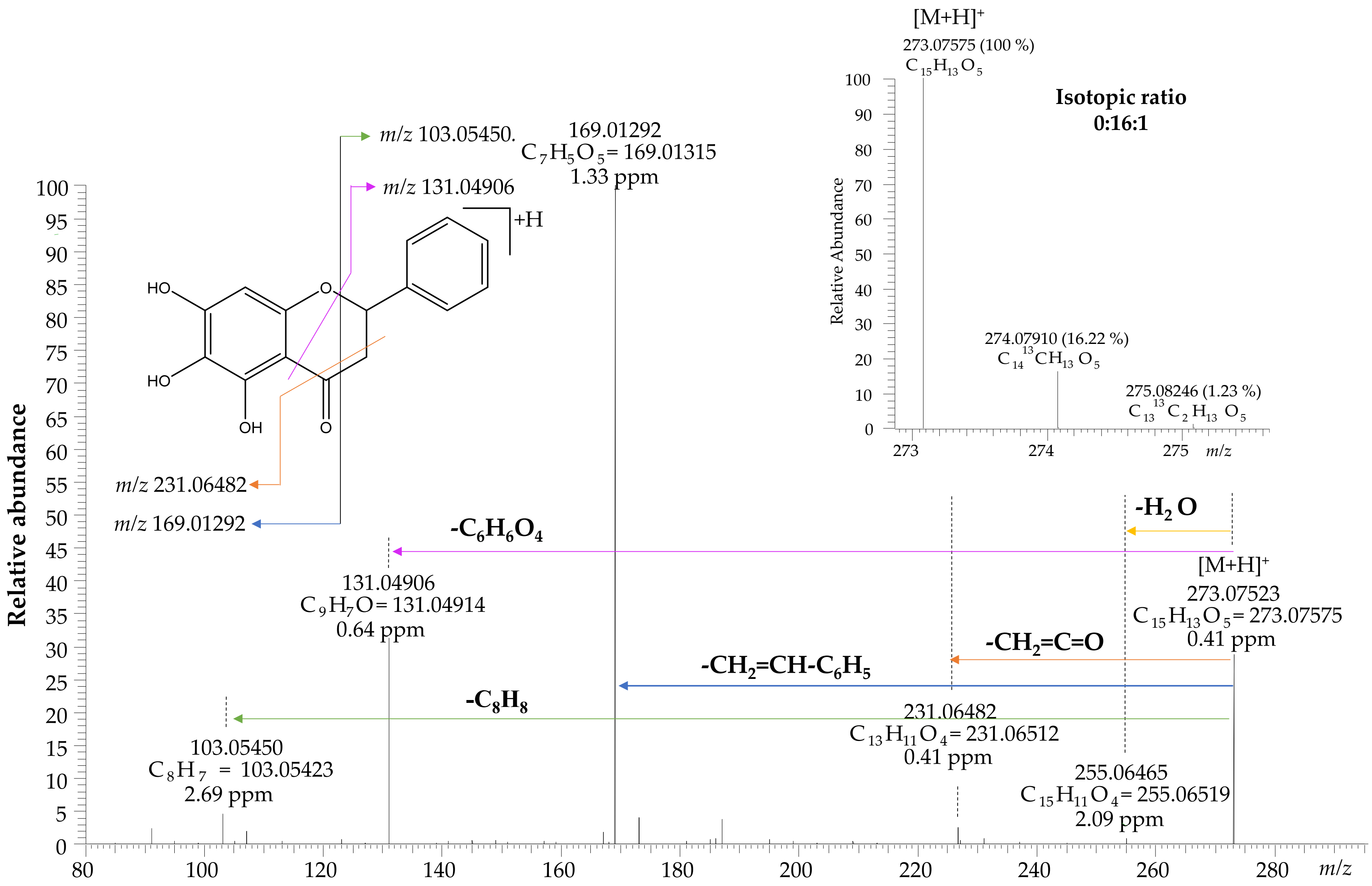

Figure S5. Mass spectrum of dihydrobaicalein, $[M+H]^+$ at *m/z* 273.07575 ($C_{15}H_{12}O_5$), present in the hydroalcoholic extract of *S. incarnata*. UHPLC/ESI-Q-Orbitrap-MS, HCD, 40 eV.

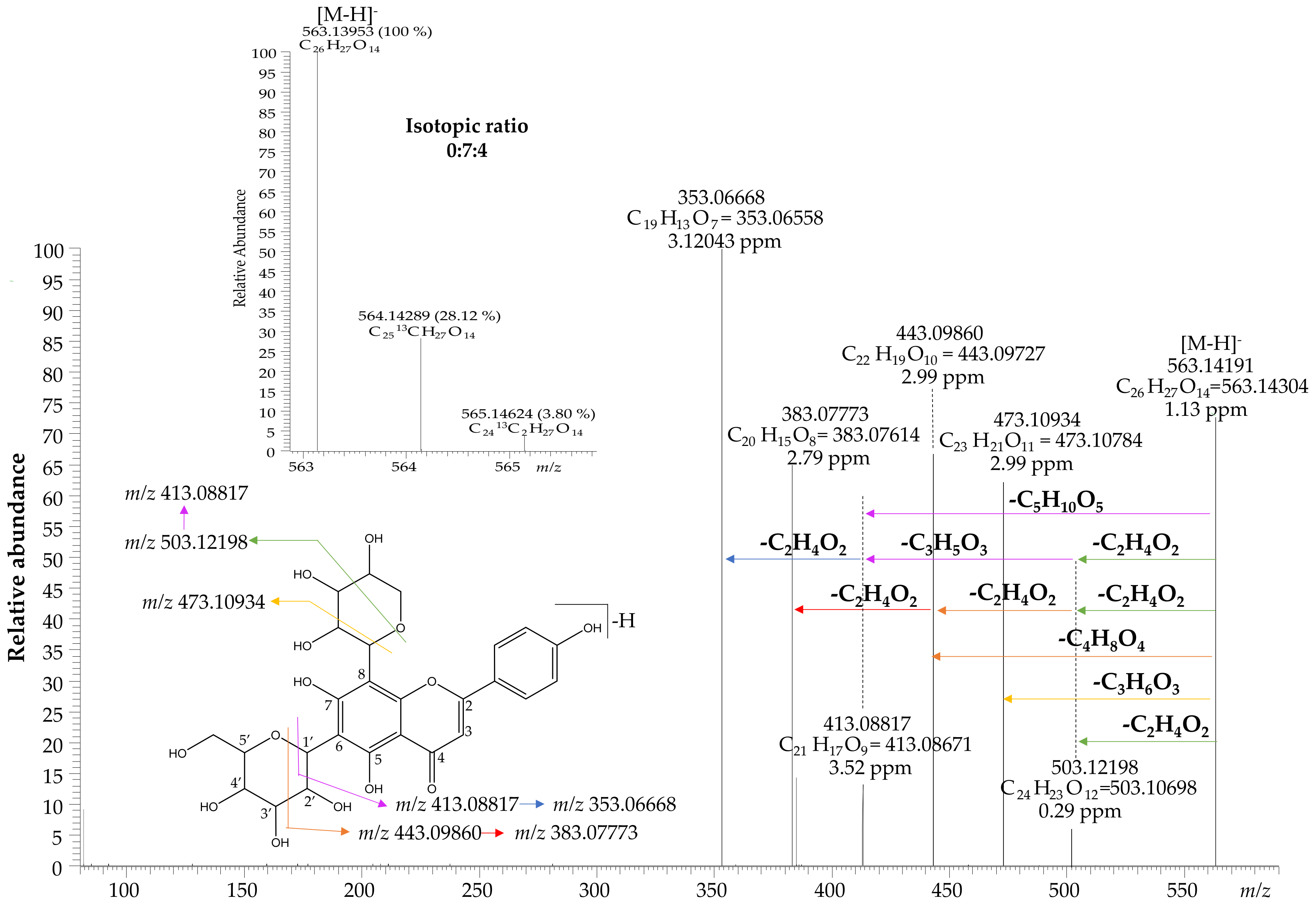

Figure S6. Mass spectrum of apigenin-*C*-glucoside-*C*-arabinoside, [M–H]- at *m/z* 563.14191 ($C_{26}H_{28}O_{14}$), present in the hydroalcoholic extract of *S. incarnata*. UHPLC/ESI-Q-Orbitrap-MS, HCD, 30 eV.

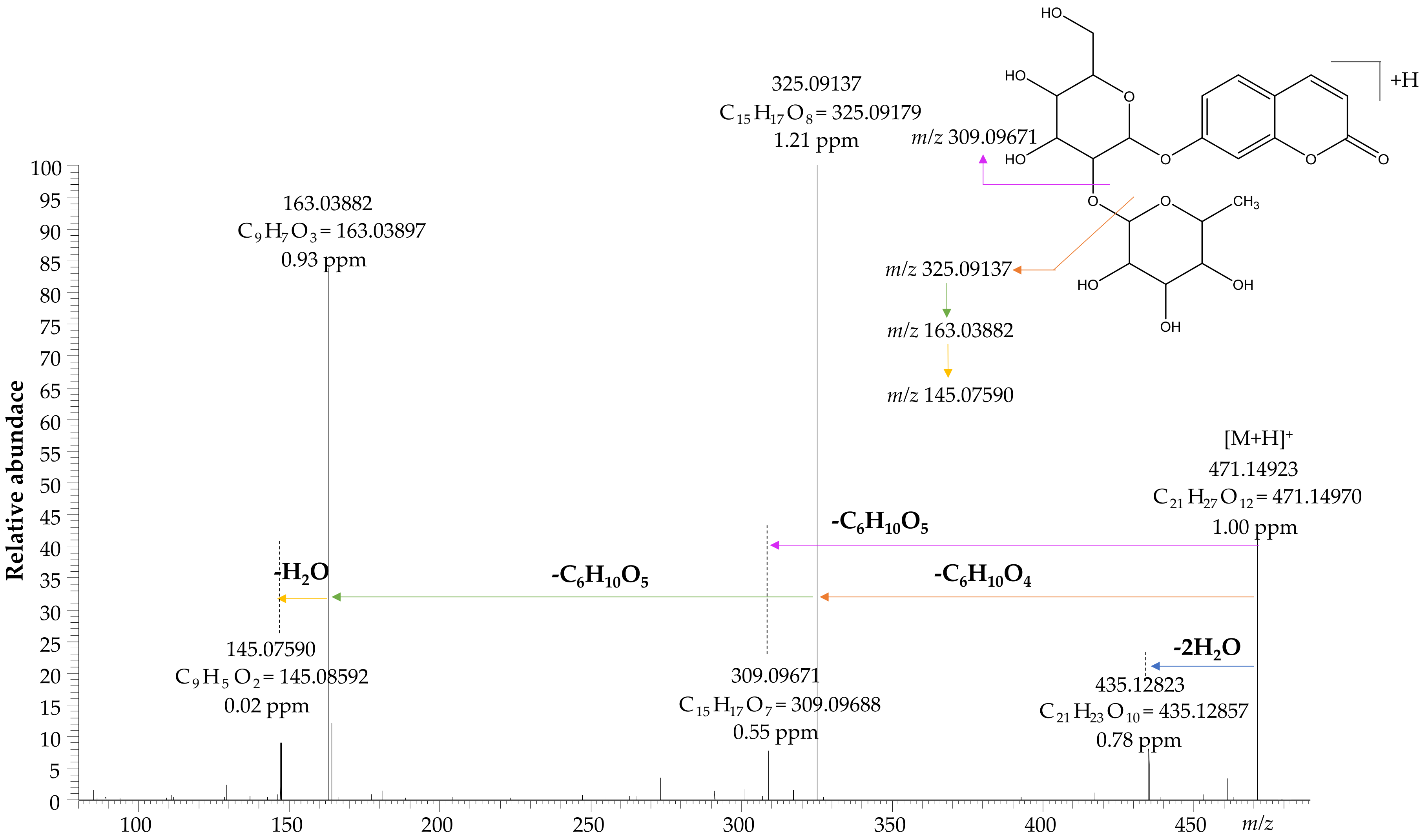

Figure S7. Mass spectrum of umbelliferone-hexoside-pentoside, [M+H]+ at *m/z* 471.14954 ($C_{21}H_{27}O_{12}$), present in the hydroalcoholic extract of *S. incarnata*. UHPLC/ESI-Q-Orbitrap-MS, HCD, 10 eV.

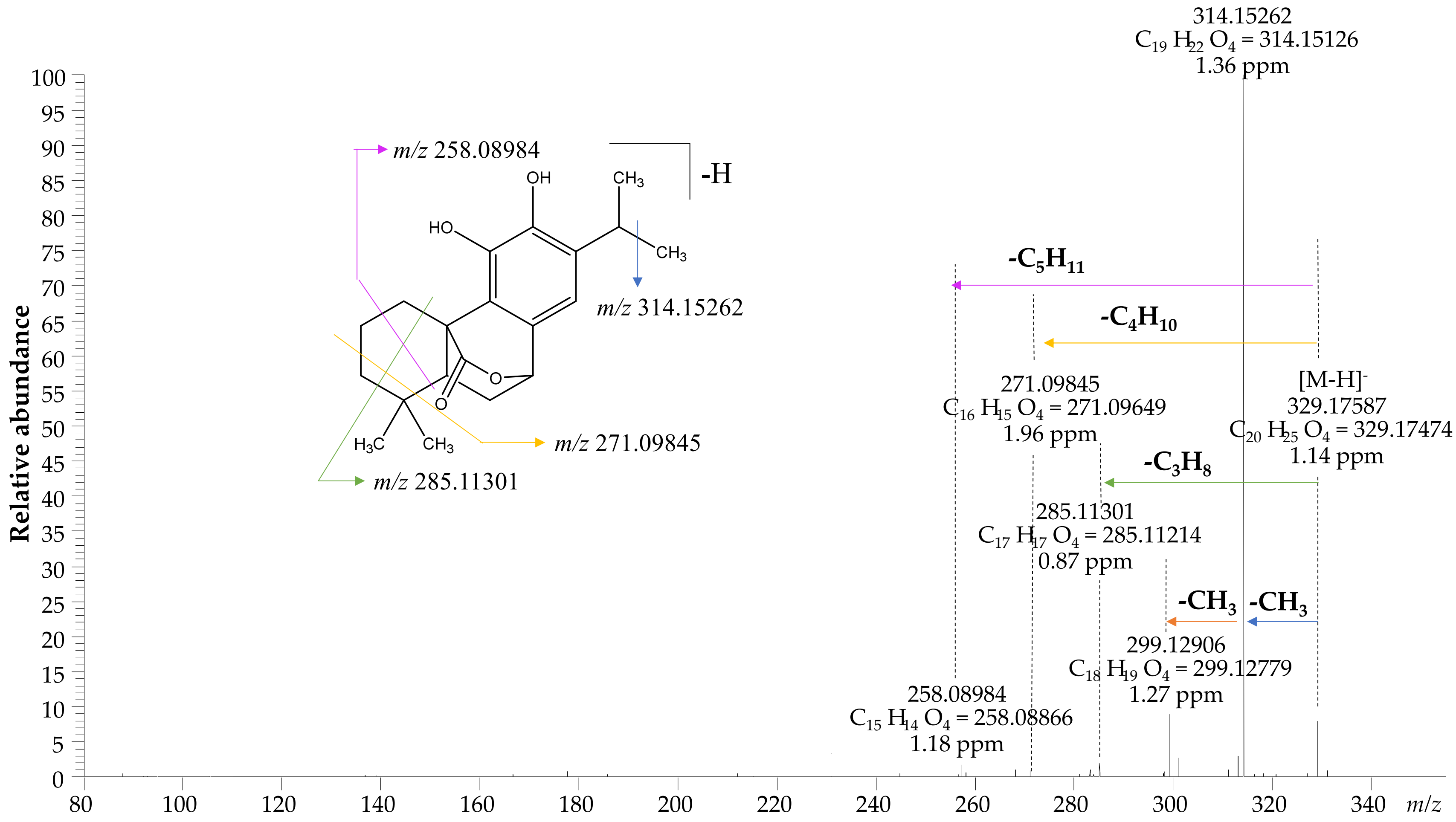

Figure S8. Mass spectrum of the unidentified compound, [M–H]$^-$ at *m/z* 329.17587 ($C_{20}H_{26}O_4$), present in the hydroalcoholic extract of *S. incarnata*. UHPLC/ESI-Q-Orbitrap-MS, HCD, 50 eV.

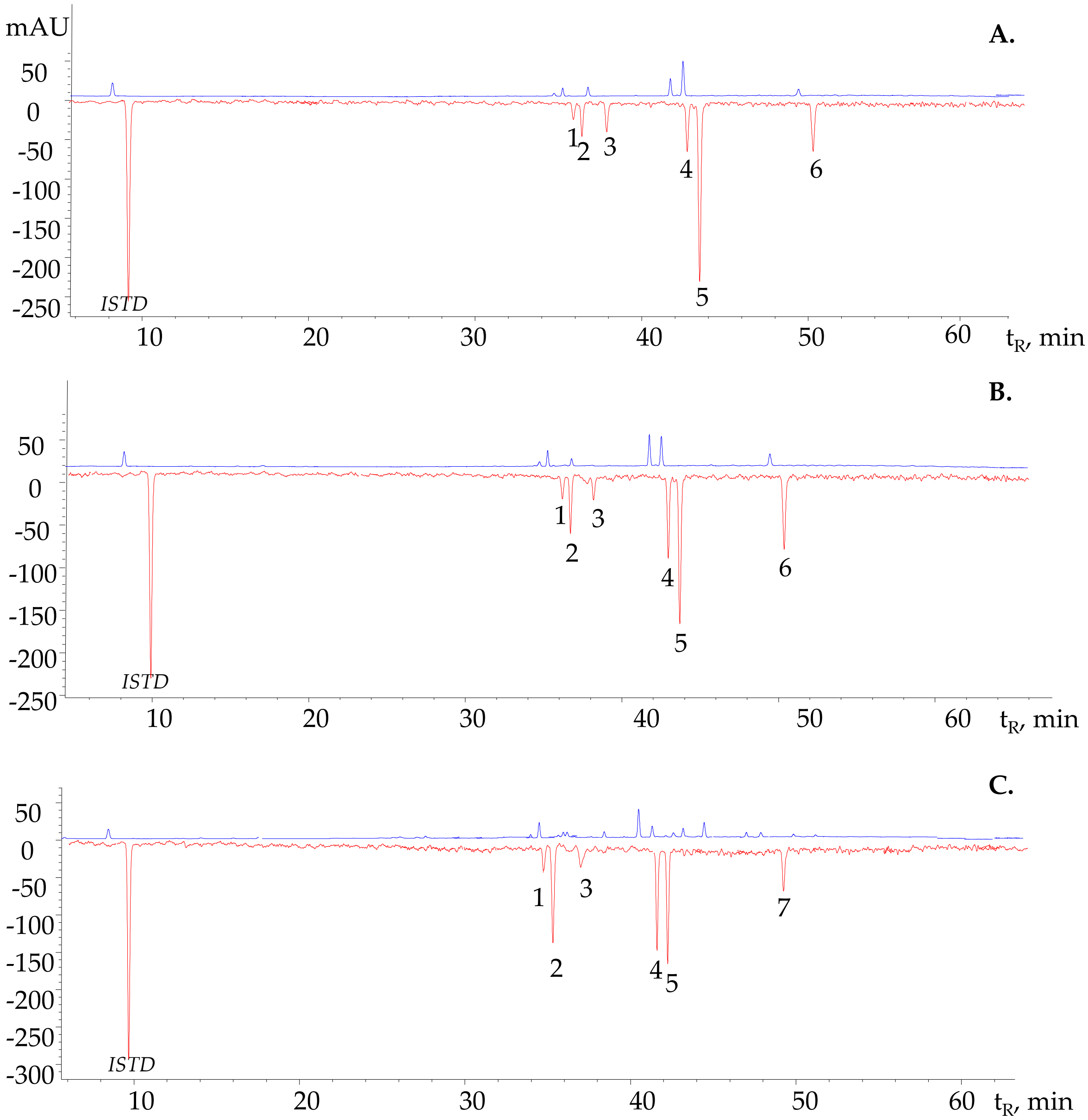

Figure S9. Chromatographic profiles obtained by on-line HPLC-ABTS$^{+•}$ of the extracts of **A.** *S. incarnata*; **B.** *S. coccinea* and **C.** *S. ventenatii* × *S. incarnata*, before (DAD, λ=290 nm, blue line) and after (MWD, λ=734 nm, red line) their reaction with the cation-radical ABTS$^{+•}$. 1. Isocarthamidin-glucuronide; 2. Verbascoside; 3. Scutellarin; 4. Baicalin; 5. Dyhydrobaicalein-glucuronide; 6. Dihydrobaicalein-baicalein; 7. Baicalein. ISTD - gallic acid (10 mg/L).

Table S2. *In vitro* antioxidant activity of the compounds present in the *Scutellaria* spp. extracts, measured by online HPLC-ABTS$^{+\bullet}$

Peak N° Fig. S9 A-C	Compounds	λ, nm	µmol Trolox®/g extract ± S, (n=2), (% of antioxidant capacity)			Ref.
			S. incarnata	*S. coccinea*	*S. ventenatii* × *S. incarnata*	
1	Isocarthamidin-glucuronide[a,b]	290[max], 340[h], 225	16.8 ± 0.9 (2 %)	19.1 ± 0.6 (2 %)	11.36 ± 0.09 (2 %)	[29]
2	Verbascoside[a,b]	330[max], 295, 230	65 ± 1 (8 %)	102.7 ± 0.6 (11 %)	119 ± 1 (24 %)	[70]
3	Scutellarin[a,b,c]	345[max], 290, 225	52 ± 2 (6 %)	48 ± 1 (5 %)	21.7 ± 0.3 (4 %)	[71]
4	Baicalin[a,b,c]	280[max], 320[h], 225	110 ± 1 (13 %)	189.1 ± 0.2 (20 %)	137 ± 2 (27 %)	[72]
5	Dihydrobaicalein-glucuronide[a,b]	280[max], 242, 363	497 ± 1 (59 %)	382 ± 1 (41 %)	163 ± 3 (32 %)	[28]
6	Dihydrobaicalein-baicalein[a,b,c]	-	101.9 ± 0.4 (12 %)	189.6 ± 0.6 (20 %)	- -	-
7	Baicalein[a,b,c]	300[max], 340[h], 230	- -	- -	51 ± 2 (10 %)	[73]

[1] Mean value ± standard deviation, n=2. [max] Maximum. [h] Shoulder.

[a] Tentative identification based on UV-Vis spectrum and comparison of typical signals λ (nm) for phenolic compounds.

[b] Tentative identification based on UV-Vis spectrum and comparison with UV- Vis spectrum reported in scientific articles [28, 29, 70-73].

[c] Tentative identification based on UV-Vis spectrum and comparison with UV- Vis spectrum and t_R of standard substance.
